# Disrupted-in-schizophrenia 1 (DISC1) and Syntaphilin collaborate to modulate axonal mitochondrial anchoring

**DOI:** 10.1186/s13041-016-0250-2

**Published:** 2016-07-02

**Authors:** Cana Park, Seol-Ae Lee, Ji-Ho Hong, Yeongjun Suh, Sung Jin Park, Bo Kyoung Suh, Youngsik Woo, Jinhyuk Choi, Ji-Won Huh, You-Me Kim, Sang Ki Park

**Affiliations:** Department of Life Sciences, Pohang University of Science and Technology, Pohang, 790-784 Republic of Korea; Division of Integrative Biosciences and Biotechnology, Pohang University of Science and Technology, Pohang, 790-784 Republic of Korea

**Keywords:** DISC1, SNPH, Axonal mitochondrial anchoring, Schizophrenia

## Abstract

In neuronal axons, the ratio of motile-to-stationary mitochondria is tightly regulated by neuronal activation, thereby meeting the need for local calcium buffering and maintaining the ATP supply. However, the molecular players and detailed regulatory mechanisms behind neuronal mitochondrial movement are not completely understood. Here, we found that neuronal activation-induced mitochondrial anchoring is regulated by Disrupted-in-schizophrenia 1 (DISC1), which is accomplished by functional association with Syntaphilin (SNPH). DISC1 deficiency resulted in reduced axonal mitochondrial movement, which was partially reversed by concomitant SNPH depletion. In addition, a SNPH deletion mutant lacking the sequence for interaction with DISC1 exhibited an enhanced mitochondrial anchoring effect than wild-type SNPH. Moreover, upon neuronal activation, mitochondrial movement was preserved by DISC1 overexpression, not showing immobilized response of mitochondria. Taken together, we propose that DISC1 in association with SNPH is a component of a modulatory complex that determines mitochondrial anchoring in response to neuronal activation.

**Electronic supplementary material:**

The online version of this article (doi:10.1186/s13041-016-0250-2) contains supplementary material, which is available to authorized users.

## Introduction

Neurons are morphologically characterized by a relatively small cell body and long extended neurites, including a single axon and varying numbers of dendrites. Neurons exhibit a localized high energy demand and a need for calcium buffering during developmental processes and synaptic functioning. These events are mainly governed by mitochondria, particularly at axon branch points and in active growth cones [1–4]. Therefore, a specialized machinery is required to distribute mitochondria to the appropriate cellular locations and to retrieve exhausted mitochondria from cellular compartments through axonal transport.

Microtubule-based axonal mitochondrial movement is largely regulated by motor and static anchor complexes. The motor-driven complex includes the kinesin-1 family (KIF) motor, the trafficking kinesin protein (TRAK), and the mitochondrial rho GTPase (MIRO) adaptor. The KIF-TRAK-MIRO complex plays a prominent role in anterograde mitochondrial movement and calcium influx-induced mitochondrial immobilization. Mitochondrial movement is facilitated by KIF coupling to the mitochondria, whereby KIF is recruited to the mitochondria by binding to TRAK, which in turn binds to the cytoplasmic surface of the mitochondria by interacting with MIRO, a transmembrane protein of the outer mitochondrial membrane. When calcium influx into neurons occurs by either activation of glutamate receptors or application of calcium ionophores, MIRO acts to sense the elevated calcium levels and inactivates or dissembles the KIF motor from the complex, resulting in mitochondrial arrest [5–8].

Syntaphilin (SNPH) is an axonally targeted static anchor protein that immobilizes mitochondria in neuronal axons. SNPH directly associates with both mitochondria and microtubules to anchor the former, thereby controlling mitochondrial transport for axonal morphogenesis and function. Cultured SNPH-deficient neurons show impaired calcium buffering in the axon terminal and enhanced mitochondrial motility together with decreased axonal branching [1]. Moreover, SNPH knockout neurons show variations in presynaptic strength, implying that arrest of axonal mitochondria by SNPH mechanistically underlies synaptic variability [9, 10]. These findings highlight the significance of SNPH-mediated mitochondrial transport in neuronal functioning, and call for further elucidation of the detailed regulatory events behind mitochondrial movement along the axon. While dynein light chain (LC8) and KIF have been documented as interaction partners for SNPH in neuronal microtubule-associated fractions [11, 12], no corresponding partners for the anchor protein have been identified in the mitochondria.

Disrupted-in-schizophrenia 1 (DISC1) is a central hub protein implicated in the pathogenesis of schizophrenia and related neurological disorders [13, 14]. DISC1 is a multifunctional molecule expressed in various neuronal compartments and functions in collaboration with assorted interaction partners [15–17]. In neuronal axons, DISC1 participates in axon guidance, synaptic development, and cargo transport [18–20]. Of particular relevance to the current study, DISC1 reportedly facilitates axonal mitochondrial movement. DISC1 physically interacts with the KIF-driven complex, but its detailed action mechanisms are not fully understood [21–23]. Here, we identified DISC1 as a novel interaction partner of SNPH that contributes to axonal mitochondrial movement and anchoring in response to neuronal activation.

## Results

### DISC1 forms a complex with SNPH

**Fig. 1:**
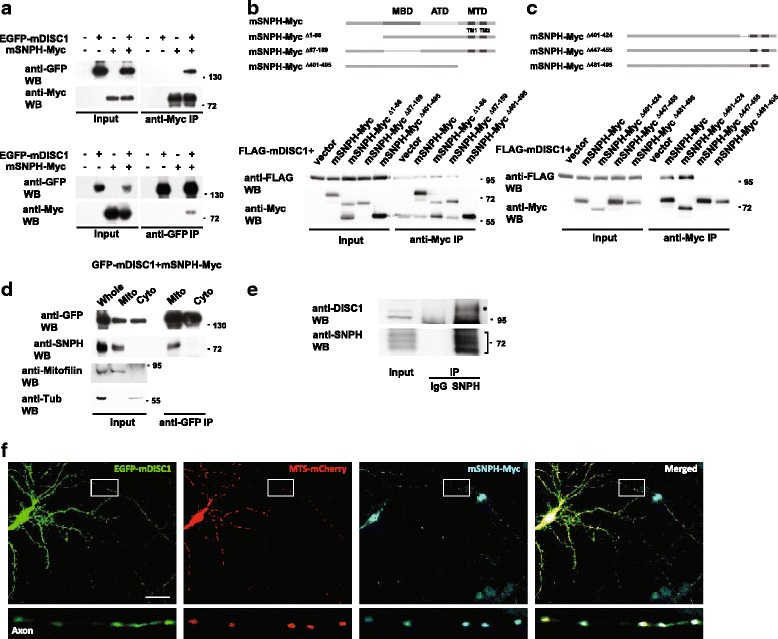
DISC1 forms a complex with SNPH. **a** Co-immunoprecipitation of DISC1 and SNPH. Lysates from CAD cells transfected with EGFP-mDISC1 and mSNPH-Myc constructs were immunoprecipitated with anti-Myc (upper) or anti-GFP (lower) antibodies. Immunoprecipitates were analyzed by western blotting with anti-GFP and anti-Myc antibodies. IP; immunoprecipitation, WB; western blotting. **b** Co-immunoprecipitation of SNPH deletion mutants and FLAG-mDISC1. A schematic diagram of SNPH domains and SNPH deletion mutants is shown. MBD; microtubule binding domain, ATD; axon targeting domain, MTD; mitochondrial transmembrane domain, TM; transmembrane segment. **c** Co-immunoprecipitation of FLAG-mDISC1 and SNPH deletion mutants in the mitochondrial transmembrane domain. A schematic diagram of SNPH deletion mutants is also shown. **d** Co-immunoprecipitation of mSNPH-Myc with EGFP-mDISC1 from the mitochondrial fraction. The mitochondrial and cytosolic fractions from CAD cells transfected with mSNPH-Myc and EGFP-mDISC1 were subjected to immunoprecipitation followed by western blotting. **e** Co-immunoprecipitation of endogenous SNPH and DISC1 from the mitochondrial fraction of mouse brain tissue. Anti-SNPH immunoprecipitates were analyzed by western blotting with anti-SNPH and anti-DISC1 antibodies. Rabbit IgG was used as negative control for immunoprecipitation. The asterisk and bracket indicate endogenous DISC1 and SNPH, respectively. **f** Co-localization of EGFP-mDISC1 and mSNPH-Myc in axon of primary cortical neurons at DIV 14 shown by immunocytochemistry. Neurons were transfected as indicated and stained with anti-Myc (*cyan*) and anti-GFP (*green*) antibodies. Scale bar; 20 μm

To identify the molecular components of the DISC1 modulatory complex for mitochondrial movement, we performed a series of co-immunoprecipitation experiments with various mitochondrial proteins. Consequently, SNPH was identified as a novel interaction partner of DISC1 (Fig. 1a).

To investigate the SNPH domain required for association with DISC1, three different SNPH domain deletion mutants that lacking the N-terminal domain, microtubule binding domain, or mitochondrial transmembrane domain were subjected to co-immunoprecipitation analysis with DISC1 (Fig. 1b). As a result, FLAG-mouse DISC1 (mDISC1) was co-immunoprecipitated with mouse SNPH (mSNPH)-Myc^∆1–86^ and mSNPH-Myc^∆87–159^, but not with mSNPH-Myc^∆401–495^. In addition, FLAG-mDISC1 failed to co-immunoprecipitate with either mSNPH-Myc^∆447–455^ or mSNPH-Myc^∆481–495^ (Fig. 1c). In case of mSNPH-Myc^∆447–455^, two mitochondrial transmembrane segments (TM1 and TM2) are directly connected by elimination of a linker sequence (amino acid residues 447–455), which is likely to disrupt the appropriate mitochondrial membrane spanning. These results indicate that DISC1 associates with the cytosolic C-terminal region of SNPH (amino acid residues 481–495).

To determine the DISC1 domain required for association with SNPH, co-immunoprecipitation using various fragments of DISC1 was performed. We found that association interface appears broadly dispersed in the DISC1 (Additional file 1: Figure S1).

We next investigated whether the potential DISC1-SNPH complex resides in mitochondria by performing co-immunoprecipitation experiments with the mitochondrial fraction of (CATH) a-differentiated (CAD) cells. Figure 1d shows that mSNPH-Myc was predominantly localized to the mitochondria and effectively co-immunoprecipitated from the mitochondrial fraction together with EGFP-mDISC1 (Fig. 1d). Importantly, endogenous DISC1 was observed in the SNPH immunoprecipitates from mitochondrial fractions and whole lysates of the mouse brain tissue, further confirming the DISC1-SNPH association in vivo (Fig. 1e and Additional file 2: Figure S2).

Furthermore, immunocytochemical analyses consistently revealed overlapping signals for EGFP-mDISC1, mSNPH-Myc, and mitochondrial transit sequence (MTS)-mCherry (a mitochondrial marker) in the axons of cortical neurons at 14 days in vitro (DIV 14) (Fig. 1f).

### The DISC1-SNPH complex regulates axonal mitochondrial movement

**Fig. 2:**
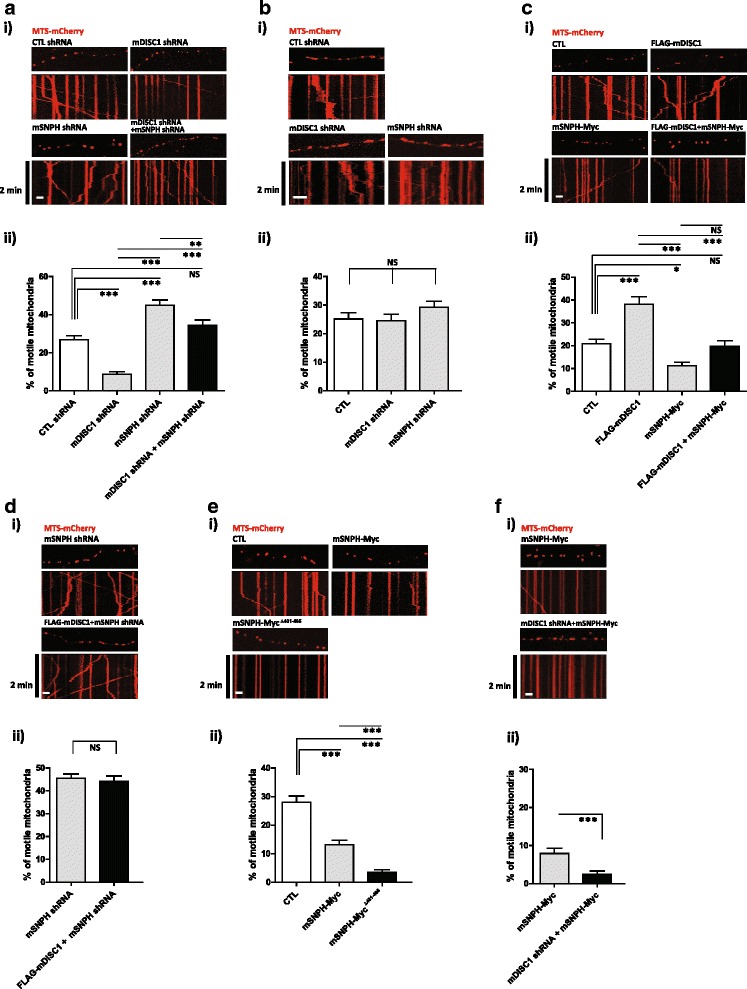
DISC1-SNPH complex regulates the mitochondrial movement in axons. **a** Reversal of DISC1 knockdown effect by SNPH knockdown. Primary cortical neurons were transfected as indicated followed by time-lapse imaging at DIV 10–14. i) Representative kymographs and ii) quantitative analyses of mitochondrial movement (*n* = 32 axons, 367 mitochondria for control shRNA, *n* = 30 axons, 385 mitochondria for mDISC1 shRNA, *n* = 32 axons, 387 mitochondria for mSNPH shRNA, and *n* = 34 axons, 397 mitochondria for mDISC1 shRNA and mSNPH shRNA). **b** Effect on dendritic mitochondrial movement. Primary cortical neurons were transfected as indicated followed by time-lapse imaging at DIV 10–14. i) Representative kymographs and ii) quantitative analyses of mitochondrial movement (*n* = 33 dendrites, 337 mitochondria for control shRNA, *n* = 31 dendrites, 392 mitochondria for mDISC1 shRNA, and *n* = 32 dendrites, 338 mitochondria for mSNPH shRNA). **c** Decreased axonal mitochondrial movement by SNPH. Primary cortical neurons were transfected as indicated followed by time-lapse imaging at DIV 10–14. i) Representative kymographs and ii) quantitative analyses of mitochondrial movement (*n* = 36 axons, 446 mitochondria for control, *n* = 34 axons, 451 mitochondria for FLAG-mDISC1, *n* = 33 axons, 428 mitochondria for mSNPH-Myc, and *n* = 37 axons, 464 mitochondria for FLAG-mDISC1 and mSNPH-Myc). **d** Effect of SNPH knockdown and DISC1 overexpression. Primary cortical neurons were transfected as indicated followed by time-lapse imaging at DIV 11. i) Representative kymographs and ii) quantitative analyses of mitochondrial movement (*n* = 34 axons, 415 mitochondria for mSNPH shRNA, and *n* = 33 axons, 432 mitochondria for FLAG-mDISC1 and mSNPH shRNA). **e** Enhanced mitochondrial anchoring by SNPH^∆481–495^. Primary cortical neurons were transfected as indicated followed by time-lapse imaging at DIV 10. i) Representative kymographs and ii) quantitative analyses of mitochondrial movement (*n* = 31 axons, 398 mitochondria for control, *n* = 31 axons, 402 mitochondria for mSNPH-Myc, and *n* = 30 axons, 392 mitochondria for mSNPH^∆481–495^-Myc). **f** Enhanced axonal mitochondrial anchoring by SNPH overexpression upon DISC1 knockdown. Primary cortical neurons were transfected as indicated followed by time-lapse imaging at DIV 10. i) Representative kymographs and ii) quantitative analyses of mitochondrial movement (*n* = 30 axons, 351 mitochondria for mSNPH-Myc, and *n* = 32 axons, 368 mitochondria for mDISC1 shRNA and mSNPH-Myc). Scale bars; 10 μm. Error bars are mean ± SEM. *P < 0.05, **P < 0.01, ***P < 0.001, NS, not significant, by student’s t-test (Fig. 2d and f) or one-way ANOVA with Bonferroni’s multiple comparison test (Fig. 2a, b, c, and e)

To analyze the functional relationship between DISC1 and SNPH in mitochondrial movement, live time-lapse imaging was employed by using primary cultured cortical neurons, as previously described [24]. Following depletion of DISC1 or SNPH with short hairpin RNA (shRNA) (Additional file 3: Figure S3), mitochondrial motility (represented by the percentage of motile mitochondria among total (motile + immobile) mitochondria) was decreased to under 10 % or increased to ~55 %, respectively (Fig. 2a, i and ii). These findings may be compared to a value of ~25 % for control (CTL) neurons and are largely consistent with previous reports [3, 22]. Moreover, the DISC1 knockdown effect was partially reversed by concomitant SNPH knockdown (Fig. 2a, i and ii). The impact of DISC1 or SNPH knockdown on mitochondrial movement was relatively specific to the axonal mitochondria, as dendritic mitochondrial motility remained largely unaffected by knockdown of either protein (Fig. 2b, i and ii).

In agreement with the above results, DISC1 overexpression increased mitochondrial motility, while SNPH overexpression exerted the opposite effect. Co-overexpression of DISC1 with wild-type SNPH did not significantly alter mitochondrial movement compared to wild-type SNPH, probably reflecting the powerful mitochondrial docking activity of wild-type SNPH (Fig. 2c, i and ii). Moreover, overexpression of DISC1 in SNPH knockdown neurons exhibited a comparable effect as SNPH knockdown, with no additional or synergistic effect (Fig. 2d, i and ii). To further characterize the significance of the DISC1-SNPH association, we employed a SNPH deletion mutant, SNPH^∆481–495^, which lacks the sequence necessary for association with DISC1. Indeed, SNPH^∆481–495^ more strongly inhibited mitochondrial motility than wild-type SNPH (Fig. 2e, i and ii). In addition, overexpression of SNPH in DISC1 knockdown neurons showed more effective mitochondrial anchoring response compared to the single overexpression SNPH effect (Fig. 2f, i and ii). These results collectively suggest that DISC1 and SNPH cooperate in the regulation of axonal mitochondrial movement.

### The DISC1-MIRO-SNPH complex mediates neuronal activation-induced mitochondrial anchoring

**Fig. 3:**
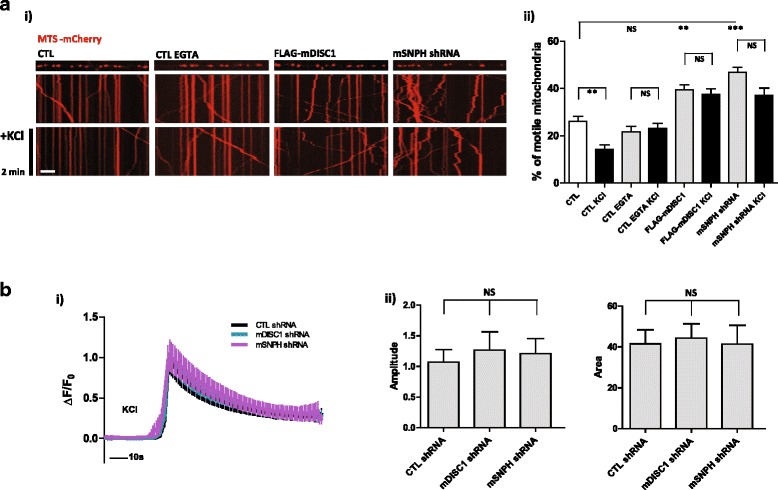
DISC1 is involved in the neuronal activation-induced mitochondrial anchoring. **a** Retained axonal mitochondrial movement upon neuronal activation by DISC1 overexpression. Primary cortical neurons were transfected as indicated followed by time-lapse imaging before and after treatment of 100 mM KCl for 5 min at DIV 10. 10 mM EGTA was treated for 20 min before KCl stimulation. Scale bar; 10 μm. i) Representative kymographs and ii) quantitative analyses were shown (*n* = 33 axons, 383 mitochondria for control, *n* = 32 axons, 395 mitochondria for control with EGTA, *n* = 34 axons, 446 mitochondria for FLAG-mDISC1, and *n* = 34 axons, 380 mitochondria for mSNPH shRNA). **b** Somatic calcium dynamics upon DISC1 or SNPH knockdown in primary cortical neurons at DIV 10. i) Calcium response graph under 100 mM KCl stimulation. ii) Statistical analyses based on peak amplitudes and average area of the graph (*n* = 43 for control shRNA, *n* = 45 for mDISC1 shRNA, and *n* = 42 for mSNPH shRNA). Error bars are mean ± SEM. *P < 0.05, **P < 0.01, ***P < 0.001, NS, not significant, by one-way ANOVA with Bonferroni’s multiple comparison test

Mitochondrial movement in neuronal axons responds to neuronal activation to properly distribute the organelles among cellular compartments, thereby meeting local calcium buffering and ATP supply demands. A recent report suggested that SNPH contributes to mitochondrial immobilization upon neuronal activation [12]. Therefore, we investigated whether DISC1 is involved in SNPH-mediated mitochondrial anchoring upon neuronal activation. Exposure of primary cortical neurons to elevated potassium chloride (KCl) levels leads to membrane depolarization and subsequent influx of calcium through L-type voltage-sensitive calcium channels [25]. Upon neuronal activation induced by 100 mM KCl for 5 min, the fraction of motile mitochondria in CTL neurons dropped to below 10 %, consistent with previous report [12]. The neurons exposed to extracellular calcium chelation with EGTA did not show any significant reduction in mitochondrial movement upon KCl stimulation, indicating the immobilized mitochondrial response is dependent upon neuronal activation-induced calcium influx. SNPH deficient neurons exhibited still retained mitochondrial movement upon neuronal activation, which is consistent with previous report [12]. Interestingly, mitochondrial motility was preserved in KCl-treated neurons that overexpressed DISC1 (Fig. 3a, i and ii).

We also assessed whether calcium influx *per se* was affected by DISC1 or SNPH expression. Because L-type calcium channels are preferentially concentrated at neuronal soma and dendrites [26, 27], we performed a somatic calcium imaging analysis by using cyto-GCaMP6s in DISC1- or SNPH-deficient neurons. KCl-stimulated somatic calcium influx responses were comparable among CTL, mDISC1 shRNA, and mSNPH shRNA groups (Fig. 3b, i and ii). These results validated the role of DISC1 and SNPH in the regulatory machinery for mitochondrial movement following neuronal activation, not affecting calcium influx.

**Fig. 4:**
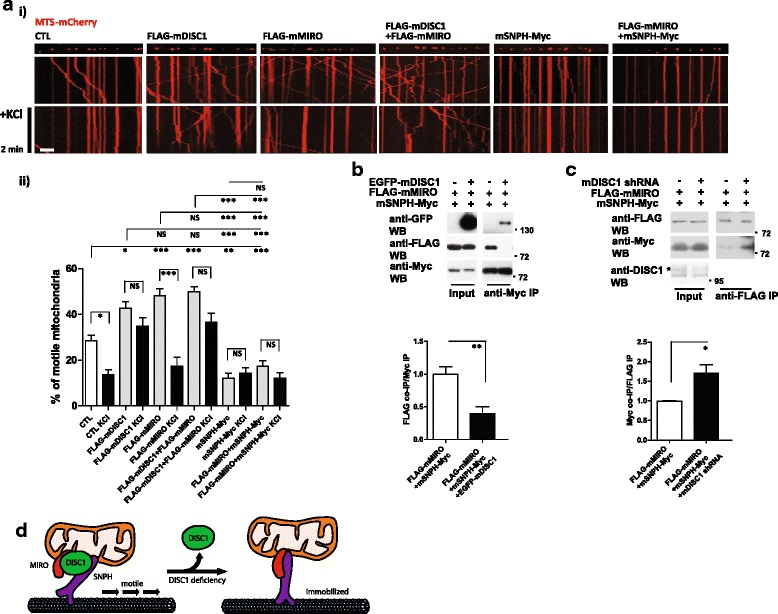
DISC1-MIRO-SNPH complex mediates the neuronal activation-induced mitochondrial anchoring. **a** Mitochondrial movement response upon neuronal activation. Primary cortical neurons were transfected as indicated followed by time-lapse imaging before and after treatment of 100 mM KCl for 5 min at DIV 10. i) Representative kymographs and ii) quantitative analyses (*n* = 30 axons, 332 mitochondria for control, *n* = 30 axons, 360 mitochondria for FLAG-mDISC1, *n* = 30 axons, 319 mitochondria for FLAG-mMIRO, *n* = 31 axons, 341 mitochondria for FLAG-mDISC1 and FLAG-mMIRO, *n* = 30 axons, 335 mitochondria for mSNPH-Myc, and *n* = 30 axons, 332 mitochondria for FLAG-mMIRO and mSNPH-Myc). Error bars are mean ± SEM. *P < 0.05, **P < 0.01, ***P < 0.001, NS, not significant, by one-way ANOVA with Bonferroni’s multiple comparison test. Scale bar; 10 μm. **b** Decreased association between SNPH and MIRO in the presence of DISC1. **c** Increased association between SNPH and MIRO in the DISC1 knockdown. Lysates from CAD cells transfected as indicated were subjected to immunoprecipitation followed by western blotting. In the western blot, the asterisk indicates the endogenous DISC1. Error bars are mean ± SEM. *P < 0.05, **P < 0.01, NS, not significant, by student’s t-test. **d** A schematic model for the roles of DISC1 in the mitochondrial motility. The association of DISC1 to SNPH inhibits the mitochondrial anchoring activity of SNPH, which facilitates the mitochondrial movement. The association between MIRO and SNPH is increased upon DISC1 deficiency, resulting in SNPH-mediated mitochondrial anchoring

We also investigated whether MIRO is responsible for DISC1-SNPH complex mediated mitochondrial anchoring upon neuronal activation and calcium influx, because MIRO is not only a calcium-responsive mitochondrial calcium sensor, but also an interaction partner of DISC1 [21, 28, 29]. Co-overexpression of DISC1 and MIRO showed no additional or synergistic effect on mitochondrial motility relative to MIRO overexpression alone. Co-overexpression of MIRO with SNPH did not reverse the strong inhibitory influence of the latter on axonal mitochondrial movement (Fig. 4a, i and ii). Of note, neurons with co-overexpressed DISC1 and MIRO still retained mitochondrial motility after KCl stimulation, as in the case of neurons with overexpressed DISC1 alone. These results differ from those of neurons with overexpressed MIRO alone, which revealed a significant decrease in motility upon neuronal activation (Fig. 4a, i and ii), potentially indicating that DISC1 regulates MIRO-mediated mitochondrial motility or regulates mitochondrial motility independent of MIRO.

Because the mitochondrial anchoring activity of SNPH is thought to be achieved by microtubule association [3], we have investigated whether SNPH or MIRO association with microtubules is affected by DISC1 using a microtubule co-sedimentation assay. We did not observe significant changes in co-sedimentation of MIRO and/or SNPH with microtubules upon the co-expression of DISC1 (Additional file 4: Figure S4). On the other hand, when we tested the associations among MIRO, DISC1, and SNPH by co-immunoprecipitation assays, FLAG-mMIRO and mSNPH-Myc showed a significant association of MIRO with SNPH (Fig. 4b). MIRO is known to associate with KIF and mediates the mitochondrial movements responding to neuronal activation [6, 28]. The association between MIRO and SNPH suggests that mitochondrial anchoring protein SNPH as well as motor protein KIF may be involved in MIRO-mediated neuronal activation-induced mitochondrial anchoring response. Importantly, the co-immunoprecipitaton of MIRO and SNPH was markedly decreased upon DISC1 overexpression (Fig. 4b), and the association was effectively reversed by DISC1 knockdown (Fig. 4c), indicating that DISC1 modulates the affinity of MIRO to the SNPH-containing mitochondrial anchoring machinery. These results potentially suggest that DISC1-SNPH complex plays an important role in mitochondrial anchoring, in which MIRO also participates.

## Discussion

The present study provides evidence for the formation of a modulatory complex by DISC1 and SNPH to regulate the ratio of stationary-to-motile mitochondria in neuronal axons. Moreover, this DISC1-SNPH complex directs the calcium influx-induced mitochondrial anchoring in response to neuronal activation, where DISC1 modulates the association between MIRO and SNPH.

DISC1 is a known component of the KIF-TRAK-MIRO complex involved in KIF motor protein-driven mitochondrial movement. In the previous study, the association of DISC1 has been demonstrated with mitochondrial movement regulators containing TRAK1 and MIRO1. TRAK1, a major axonal mitochondrial protein, increases mitochondrial DISC1 content, in turn enhancing the association of KIF with the mitochondrial membrane and KIF-facilitated anterograde axonal mitochondrial movement [21]. More recently, DISC1 is also shown to interact with the dendritic mitochondrial transport protein, TRAK2, to regulate dendritic mitochondrial motility. Moreover, DISC1 forms a complex with the mitofusins, a group of regulators of mitochondrial dynamics. Along the line, DISC1-Boymaw fusion protein, induces abnormalities not only in mitochondrial movement, but also in mitochondrial dynamics [30–32]. These findings indicate that DISC1-mediated mitochondrial movement is normally linked to mitochondrial biogenesis and dynamics. Additionally, our findings demonstrate that DISC1 associates with the mitochondrial anchoring protein, SNPH and that the DISC1-SNPH complex contributes to neuronal activation-induced mitochondrial immobilization in axons. DISC1 weakens the association between MIRO and SNPH, supposedly resulting in dissociation of mitochondrial anchoring by SNPH from neuronal activation-induced calcium influx sensed by MIRO. Based on these findings, we propose that DISC1 plays modulatory roles for axonal mitochondrial movement and anchoring at the interface of KIF-TRAK-MIRO and SNPH-MIRO complexes upon changes in axonal physiology.

Lines of evidence indicate that altered axonal mitochondrial distribution contributes to the pathogenesis of mental illness including schizophrenia. Post-mortem studies of schizophrenic patients revealed similar overall numbers of mitochondria; however, the axonal distribution of mitochondria differed in the caudate nucleus and putamen, with fewer mitochondria per synapse than normal [33]. Likewise, axonal termini exhibited a decreased mitochondrial density in the post-mortem anterior cingulate cortex of the schizophrenic brain [34]. Drug-naïve patients with schizophrenia also showed a reduced mitochondrial density in axon termini, whereas this phenomenon was not seen in patients taking antipsychotic medications [35]. These results indicate that abnormalities in intracellular distribution of mitochondria are associated with disorders. In the related line, the potential roles of DISC1-SNPH complex in abnormal distribution of mitochondria associated with specific clinical conditions would be of immediate interest.

Recent studies indicated that DISC1 physically interacts with amyloid precursor protein (APP) and regulates neuronal migration and neurite outgrowth [36]. DISC1 also directly affects C-terminal processing of APP and generation of the β-amyloid (Aβ) peptide, a pathological hallmark of Alzheimer’s disease (AD) [37]. These findings suggest that two notable high risk factors of neurological diseases, DISC1 and APP, jointly rather than independently affect neuropathological processes. Strikingly, brief exposure of cultured hippocampal neurons to the Aβ peptide results in the acute and severe impairment of mitochondrial transport [38]. The protein kinase A/glycogen synthase kinase 3β signal transduction cascades, a major DISC1 regulatory pathway, are reportedly involved in this phenomenon [38, 39]. Therefore, the potential link between DISC1 and APP in the context of axonal mitochondrial transport demands further investigation.

## Methods

### Plasmids and antibodies

For mouse SNPH (mSNPH) construct, an isoform of mSNPH coding sequence (NM_001291076.1) was amplified from the cDNA library obtained from primary cultured neurons and inserted into pCDNA3.1 Myc-His. For construct of MIRO, a mouse MIRO1 (mMIRO) from the cDNA library was amplified and inserted into pFLAG-CMV2. For construct of MTS-mCherry, mitochondrial transit sequence (MTS) of human COXVII was inserted into pEGFP-N1, in which EGFP was substituted to mCherry. The cyto-GCaMP6s construct was purchased from Addgene. To construct the deletion mutants of SNPH, the regions of mSNPH corresponding to the designated codons were amplified by PCR using mSNPH-Myc as a template and inserted into the pCDNA3.1 Myc-His. Sequences were verified by DNA sequencing. The mDISC1 shRNA was used as previously described [16]. The oligonucleotide sequences for mSNPH shRNA construct are 5′-TGAACAACCTGATTGACAAGGACTTCAAGAGAGTCCTTGTCAATCAGGTTGTTCTTTTTTC-3′ and 5′-TCGAGAAAAAAGAACAACCTGATTGACAAGGACTCTCTTGAAGTCCTTGTCAATCAGGTTGTTCA-3′. These oligonucleotides were annealed and ligated into the pLentiLox3.7 vector using Hpa I and Xho I sites. Anti-GFP (A11122) antibody was purchased from Molecular Probes. Anti-FLAG M2 (F1804) antibody were purchased from Sigma. Anti-FLAG (PA1-984B) antibody was purchased from Thermo Fisher Scientific. Anti-Myc (9E10) antibody was purchased from Santa Cruz Biotechnology. Anti-SNPH (13646-1-AP) and anti-Tubulin (66031-1-Ig) antibodies were purchased from Proteintech. Anti-mitofilin (NB00-1919) antibody was purchased from NOVUS Biologicals. Anti-DISC1 (ABN425) antibody was purchased from Millipore.

### Mouse line

Pregnant Institute for Cancer Research (ICR, CrljBgi:CD-1) mice were purchased from Hyochang Science and embryos were processed for the culture of primary neurons. Dr. Kaibuchi (Nagoya University) kindly provided the whole brain lysate from DISC1 mutant mice lacking DISC1 exons 2 and 3 [40].

### Transfection and mitochondrial fractionation

(CATH) a-differentiated (CAD) or HEK293 cells were grown in DMEM supplemented with 10 % fetal bovine serum and antibiotics under 5 % CO_2_ at 37 °C. Cells were transfected using VivaMagic (Vivagen) or Lipofectamine 2000 (Invitrogen) according to the manufacturer’s instruction. Harvested cells or ICR mouse brain tissue were washed with PBS, suspended in a mitochondria isolation buffer (250 mM sucrose, 1 mM EGTA, 1 mM MgCl_2_, 0.5 mM DTT, 10 mM Tris, pH 8.0), and disrupted by dounce homogenization. The homogenate was spun at 800 × g for 10 min. The supernatant was recovered and centrifuged again for 10 min at 8,000 × g. The resulting pellet (mitochondrial fraction) was collected, while the supernatant (cytosolic fraction) was cleared by further centrifugation for 10 min at 12,000 × g.

### Co-immunoprecipitation

Cultured cells, mitochondrial fractions isolated from CAD cells, or ICR mouse brain tissue were homogenized in ELB lysis buffer (50 mM Tris, pH 8.0, 250 mM NaCl, 0.1 % NP-40, 5 mM EDTA, 5 mM glycerol-2-phosphate, 2 mM sodium pyrophosphate, 5 mM NaF, 2 mM Na_3_VO_4_, 1 mM DTT, EDTA-free protease inhibitor cocktail) and pre-cleared by centrifugation for 10 min at 12,000 × g. For immunoprecipitation, the lysates were incubated with antibody (0.5–1 μg) on a rocking platform for overnight at 4 °C, and then 20 μl of 10 % protein-A agarose (Roche) resuspended in the same lysis buffer was added and incubated with gentle shaking for an additional 2–3 h at 4 °C. The precipitates were washed three times with lysis buffer and resuspended in the SDS sample loading buffer. Precipitates were subjected to anti-DISC1, anti-SNPH, anti-GFP, anti-Myc, or anti-FLAG western blotting.

### Immunocytochemistry

Primary cortical neurons at DIV 14 were fixed for 15 min in 4 % paraformaldehyde in PBS and incubated for 20 min in the blocking solution (2 % goat serum and 1 % Triton X-100 in PBS). Neurons were incubated with mouse anti-Myc (1:100, Santa Cruz) and rabbit anti-GFP antibodies (1:2000, Molecular Probes) for 3 h at room temperature, followed by incubation with Alexa Fluor 488-conjugated goat anti-rabbit IgG and Alexa Fluor 647-conjugated goat anti-mouse IgG secondary antibodies (Invitrogen) for 2 h at room temperature. The images were obtained using laser scanning confocal microscope (Olympus, FluoView-1000).

### Primary cortical neuron culture and mitochondrial movement assay

Developing cortices were dissected from embryonic day 15–16 (E15-16) ICR mice in Hank’s balanced salt solution (HBSS, Invitrogen). Dissected tissues were dissociated by treating with DNase I (0.1 %) and Trypsin (0.25 %) for 15 min at 37 °C. The dissociated cells were diluted in plating media, neurobasal media (Invitrogen) containing 10 mM HEPES (pH 7.4) and 10 % horse serum (Gibco), and then plated onto the glass bottom dish (ibidi) coated with poly-D-lysine and laminin. Plating media was replaced with culture media, neurobasal media supplemented with 2 % B27 (Invitrogen), 2 mM glutamine and antibiotics. At DIV 7–10, plasmid DNA constructs as indicated were transfected with Lipofectamine 2000. At DIV 10–14 (day 3 or day 4 post-transfection), live time-lapse imaging was performed using spinning disc confocal microscope (Olympus, IX81) following previous description with modifications [24]. During imaging, the neurons were transferred to the microscope live-cell chamber maintaining 37 °C and 5 % CO_2._ Neurons were imaged for 2 min with 10 s interval. The obtained images were subjected to the analyses for the mitochondrial motility as previously described [24]. Axonal mitochondria were primarily analyzed for the mitochondrial movement assay. Based on the morphological criteria, an axon was identified as a long and thin process while dendrites were shorter and thicker. In axons, a frame with average 150 μm length from at least 100 μm away from soma was selected and analyzed. In dendrites of neuron, the longest dendrite was selected and a frame average 100 μm length from 50 μm away from soma was subject to the analysis. Mitochondrion showing a displacement from the original point at least 2 μm for 2 min was regarded as motile one. The motile and stationary mitochondria were counted manually based on the image sequences and kymographs generated. The movement of mitochondria was presented as the percentage of motile mitochondria. Sample size was more than 30 axons or dendrites. The number of mitochondria analyzed is described in the figure legends.

### Calcium imaging

Live calcium imaging was performed following previous descriptions [41, 42]. Cultured cortical neurons at DIV 7 from ICR mouse embryos were transfected with shRNA constructs and cyto-GCaMP6s followed by calcium imaging at DIV 10. Primary cultured neurons were loaded with low potassium buffer (10 mM HEPES, pH 7.4, 126 mM NaCl, 4 mM KCl, 2 mM CaCl_2_, 1 mM MgCl_2_, 4.2 mM glucose) and treated with 100 mM KCl. The fluorescence intensities were recorded in Metamorph software at an interval of 1 s using spinning disc confocal microscope (Olympus, IX81).

### Microtubule co-sedimentation assay

Microtubule co-sedimentation assay was performed following previous descriptions with modifications [11]. Cultured HEK293 cells were lysed in 80 mM PIPES (pH 7.0), 1 % Triton X-100, 1 mM MgCl_2_, 1 mM EGTA, 1 mM phenylmethylsulfonyl fluoride (PMSF), 0.5 mM vanadate, and protease inhibitor cocktail. After incubation on ice for 30 min to depolymerize the microtubules, the lysate was centrifuged at 20,000 × g for 40 min 4 °C to remove cellular debris. The supernatant was treated with 20 μM Taxol for 30 min at 37 °C, followed by centrifugation at 12,000 × g for 40 min at room temperature. The resultant pellets resuspended in lysis buffer and supernatant were subjected to the western blotting.

### Statistical analysis

Data were obtained at least three independent experiments. Numbers of cells studied are given in the figure legends. Data were analyzed using the GraphPad Prism 5 software and presented as the mean ± SEM. Statistical significance was determined by student’s t-test or one-way ANOVA with Bonferroni’s multiple comparison test.

## Abbreviations

APP, amyloid precursor protein; Aβ, β-amyloid; CAD, (CATH) a-differentiated; DISC1, disrupted-in-schizophrenia 1; EGTA, ethylene glycol tetraacetic acid; KCl, potassium chloride; KIF, kinesin-1 family; LC, light chain; MIRO, mitochondrial rho GTPase; MTS, mitochondrial transit sequence; shRNA, short hairpin RNA; SNPH, syntaphilin; TRAK, trafficking kinesin protein

## Additional files

Additional file 1: Figure S1. Multiple SNPH association regions of DISC1. (A) Co-immunoprecipitation of FLAG-mDISC1 fragments with mSNPH-Myc in HEK293 cells. Lysates were immunoprecipitated with anti-FLAG and subjected to anti-FLAG and anti-SNPH western blotting. (TIF 1174 kb) Additional file 2: Figure S2. DISC1 and SNPH association in the mouse whole brain tissue. (A) Co-immunoprecipitation of endogenous SNPH and DISC1 from the mouse brain lysates. Anti-SNPH immunoprecipitates were analyzed by western blotting with anti-SNPH and anti-DISC1 antibodies. The asterisk and bracket indicate endogenous DISC1 and SNPH, respectively. (TIF 674 kb) Additional file 3: Figure S3. Characterization of shRNA constructs and antibodies against DISC1 and SNPH. (A) Knockdown of endogenous DISC1 in the differentiated CAD cells using DISC1 shRNA. Quantification of band intensities is also shown. Error bars represent means ± SEM. *** *P* < 0.001 (student’s t-test, *n* = 3). (B) Characterization of DISC1 antibody in the whole brain lysates. (i) Detection of DISC1 in the brain lysates from the wild-type (WT) and mutant mice lacking exons 2 and 3 of *Disc1* gene [*Disc1* (∆ 2–3)]. (ii) The immunoprecipitated DISC1 from mouse brain lysates. The asterisk indicates the endogenous DISC1 band. (C) Knockdown of overexpressed GFP-mSNPH (i) and endogenous SNPH (ii) in the differentiated CAD cells using SNPH shRNA. GFP-mSNPH is indicated by a single asterisk in the western blotting. Quantification of band intensities is also shown. Error bars represent means ± SEM. **P* < 0.05, ***P* < 0.01 (student’s t-test, *n* = 3). (TIF 1040 kb) Additional file 4: Figure S4. Unaffected microtubule association of SNPH and MIRO upon DISC1 co-expression. (A) Microtubule co-sedimentation assay. HEK293 cell lysates transfected as indicated were incubated with 20 μM of Taxol for 30 min at 37 °C. Following the sedimentation of polymerized microtubule by centrifugation at 12,000 g for 40 min at room temperature, same amount of each sample were subjected to the (i) western blotting and (ii) densitometric analysis S; supernatant. P; pellet. (TIF 725 kb)

## References

[1] Courchet J, Lewis TL, Lee S, Courchet V, Liou DY, Aizawa S, Polleux F (2013). Terminal axon branching is regulated by the LKB1-NUAK1 kinase pathway via presynaptic mitochondrial capture. Cell.

[2] Ruthel G, Hollenbeck PJ (2003). Response of mitochondrial traffic to axon determination and differential branch growth. J Neurosci.

[3] Kang JS, Tian JH, Pan PY, Zald P, Li C, Deng C, Sheng ZH (2008). Docking of axonal mitochondria by syntaphilin controls their mobility and affects short-term facilitation. Cell.

[4] Lee D, Michalak M (2012). Calcium and bioenergetics: from endoplasmic reticulum to mitochondria. Anim Cells Syst.

[5] Sheng ZH, Cai Q (2012). Mitochondrial transport in neurons: impact on synaptic homeostasis and neurodegeneration. Nat Rev Neurosci.

[6] MacAskill AF, Rinholm JE, Twelvetrees AE, Arancibia-Carcamo IL, Muir J, Fransson A, Aspenstrom P, Attwell D, Kittler JT (2009). Miro1 Is a Calcium Sensor for Glutamate Receptor-Dependent Localization of Mitochondria at Synapses. Neuron.

[7] Fujita T, Maturana AD, Ikuta J, Hamada J, Walchli S, Suzuki T, Sawa H, Wooten MW, Okajima T, Taternatsu K (2007). Axonal guidance protein FEZ1 associates with tubulin and kinesin motor protein to transport mitochondria in neurites of NGF-stimulated PC12 cells. Biochem Bioph Res Co.

[8] van Spronsen M, Mikhaylova M, Lipka J, Schlager MA, van den Heuve DJ, Kuijpers M, Wulf PS, Keijzer N, Demmers J, Kapitein LC (2013). TRAK/Milton Motor-Adaptor Proteins Steer Mitochondrial Trafficking to Axons and Dendrites. Neuron.

[9] Sun T, Qiao H, Pan PY, Chen Y, Sheng ZH (2013). Motile axonal mitochondria contribute to the variability of presynaptic strength. Cell Rep.

[10] Murthy VN, Sejnowski TJ, Stevens CF (1997). Heterogeneous release properties of visualized individual hippocampal synapses. Neuron.

[11] Chen YM, Gerwin C, Sheng ZH (2009). Dynein light chain LC8 regulates syntaphilin-mediated mitochondrial docking in axons. J Neurosci Off J Soc Neurosci.

[12] Chen Y, Sheng ZH (2013). Kinesin-1-syntaphilin coupling mediates activity-dependent regulation of axonal mitochondrial transport. J Cell Biol.

[13] Millar JK, Wilson-Annan JC, Anderson S, Christie S, Taylor MS, Semple CAM, Devon RS, St Clair DM, Muir WJ, Blackwood DHR (2000). Disruption of two novel genes by a translocation co-segregating with schizophrenia. Hum Mol Genet.

[14] Brandon NJ, Sawa A (2011). Linking neurodevelopmental and synaptic theories of mental illness through DISC1. Nat Rev Neurosci.

[15] Morris JA, Kandpal G, Ma L, Austin CP (2003). DISC1 (Disrupted-In-Schizophrenia 1) is a centrosome-associated protein that interacts with MAP1A, MIPT3, ATF4/5 and NUDEL: regulation and loss of interaction with mutation. Hum Mol Genet.

[16] Park YU, Jeong J, Lee H, Mun JY, Kim JH, Lee JS, Nguyen MD, Han SS, Suh PG, Park SK (2010). Disrupted-in-schizophrenia 1 (DISC1) plays essential roles in mitochondria in collaboration with Mitofilin. Proc Natl Acad Sci U S A.

[17] Shimizu S, Matsuzaki S, Hattori T, Kumamoto N, Miyoshi K, Katayama T, Tohyama M (2008). DISC1-kendrin interaction is involved in centrosomal microtubule network formation. Biochem Bioph Res Co.

[18] Chen SY, Huang PH, Cheng HJ (2011). Disrupted-in-Schizophrenia 1-mediated axon guidance involves TRIO-RAC-PAK small GTPase pathway signaling. Proc Natl Acad Sci U S A.

[19] Faulkner RL, Jang MH, Liu XB, Duan X, Sailor KA, Kim JY, Ge S, Jones EG, Ming GL, Song HJ (2008). Development of hippocampal mossy fiber synaptic outputs by new neurons in the adult brain. Proc Natl Acad Sci U S A.

[20] Kittler J (2013). The Role of DISC1 in the Intracellular Transport of Mitochondria and Other Cargo. Biol Psychiat.

[21] Ogawa F, Malavasi EL, Crummie DK, Eykelenboom JE, Soares DC, Mackie S, Porteous DJ, Millar JK (2014). DISC1 complexes with TRAK1 and Miro1 to modulate anterograde axonal mitochondrial trafficking. Hum Mol Genet.

[22] Atkin TA, MacAskill AF, Brandon NJ, Kittler JT (2011). Disrupted in Schizophrenia-1 regulates intracellular trafficking of mitochondria in neurons. Mol Psychiatry.

[23] Atkin TA, Brandon NJ, Kittler JT (2012). Disrupted in Schizophrenia 1 forms pathological aggresomes that disrupt its function in intracellular transport. Hum Mol Genet.

[24] Wang XN, Schwarz TL (2009). Imaging Axonal Transport of Mitochondria. Method Enzymol.

[25] Malik AN, Vierbuchen T, Hemberg M, Rubin AA, Ling E, Couch CH, Stroud H, Spiegel I, Farh KK, Harmin DA (2014). Genome-wide identification and characterization of functional neuronal activity-dependent enhancers. Nat Neurosci.

[26] Hell JW, Westenbroek RE, Warner C, Ahlijanian MK, Prystay W, Gilbert MM, Snutch TP, Catterall WA (1993). Identification and differential subcellular localization of the neuronal class C and class D L-type calcium channel alpha 1 subunits. J Cell Biol.

[27] Simon M, Perrier JF, Hounsgaard J (2003). Subcellular distribution of L-type Ca2+ channels responsible for plateau potentials in motoneurons from the lumbar spinal cord of the turtle. Eur J Neurosci.

[28] Liu XG, Hajnoczky G (2009). Ca2 + −dependent regulation of mitochondrial dynamics by the Miro-Milton complex. Int J Biochem Cell B.

[29] Wang XN, Schwarz TL (2009). The Mechanism of Ca2 + −Dependent Regulation of Kinesin-Mediated Mitochondrial Motility. Cell.

[30] Zhou X, Chen Q, Schaukowitch K, Kelsoe JR, Geyer MA (2010). Insoluble DISC1-Boymaw fusion proteins generated by DISC1 translocation. Mol Psychiatry.

[31] Norkett R, Modi S, Birsa N, Atkin TA, Ivankovic D, Pathania M, Trossbach SV, Korth C, Hirst WD, Kittler JT (2016). DISC1-dependent Regulation of Mitochondrial Dynamics Controls the Morphogenesis of Complex Neuronal Dendrites. J Biol Chem.

[32] Ji B, Higa KK, Kim M, Zhou L, Young JW, Geyer MA, Zhou X (2014). Inhibition of protein translation by the DISC1-Boymaw fusion gene from a Scottish family with major psychiatric disorders. Hum Mol Genet.

[33] Somerville SM, Conley RR, Roberts RC (2011). Mitochondria in the striatum of subjects with schizophrenia. The World Journal of Biological Psychiatry: The Official Journal of the World Federation of Societies of Biological Psychiatry.

[34] Roberts RC, Barksdale KA, Roche JK, Lahti AC (2015). Decreased synaptic and mitochondrial density in the postmortem anterior cingulate cortex in schizophrenia. Schizophr Res.

[35] Kung L, Roberts RC (1999). Mitochondrial pathology in human schizophrenic striatum: a postmortem ultrastructural study. Synapse.

[36] Young-Pearse TL, Suth S, Luth ES, Sawa A, Selkoe DJ (2010). Biochemical and functional interaction of disrupted-in-schizophrenia 1 and amyloid precursor protein regulates neuronal migration during mammalian cortical development. J Neurosci Off J Soc Neurosci.

[37] Shahani N, Seshadri S, Jaaro-Peled H, Ishizuka K, Hirota-Tsuyada Y, Wang Q, Koga M, Sedlak TW, Korth C, Brandon NJ (2015). DISC1 regulates trafficking and processing of APP and Abeta generation. Mol Psychiatry.

[38] Rui Y, Tiwari P, Xie Z, Zheng JQ (2006). Acute impairment of mitochondrial trafficking by beta-amyloid peptides in hippocampal neurons. J Neurosci Off J Soc Neurosci.

[39] Ishizuka K, Kamiya A, Oh EC, Kanki H, Seshadri S, Robinson JF, Murdoch H, Dunlop AJ, Kubo K, Furukori K (2011). DISC1-dependent switch from progenitor proliferation to migration in the developing cortex. Nature.

[40] Kuroda K, Yamada S, Tanaka M, Iizuka M, Yano H, Mori D, Tsuboi D, Nishioka T, Namba T, Iizuka Y (2011). Behavioral alterations associated with targeted disruption of exons 2 and 3 of the Disc1 gene in the mouse. Hum Mol Genet.

[41] Bootman MD, Rietdorf K, Collins T, Walker S, Sanderson M (2013). Ca2 + −sensitive fluorescent dyes and intracellular Ca2+ imaging. Cold Spring Harb Protoc.

[42] Grienberger C, Konnerth A (2012). Imaging calcium in neurons. Neuron.

