# Supplementary figures and images for: Disrupted-in-schizophrenia 1 (DISC1) and Syntaphilin collaborate to modulate axonal mitochondrial anchoring
Source: Mol Brain. 2016 Jul 2;9:69. doi: 10.1186/s13041-016-0250-2 (PMC4930613; doi:10.1186/s13041-016-0250-2)

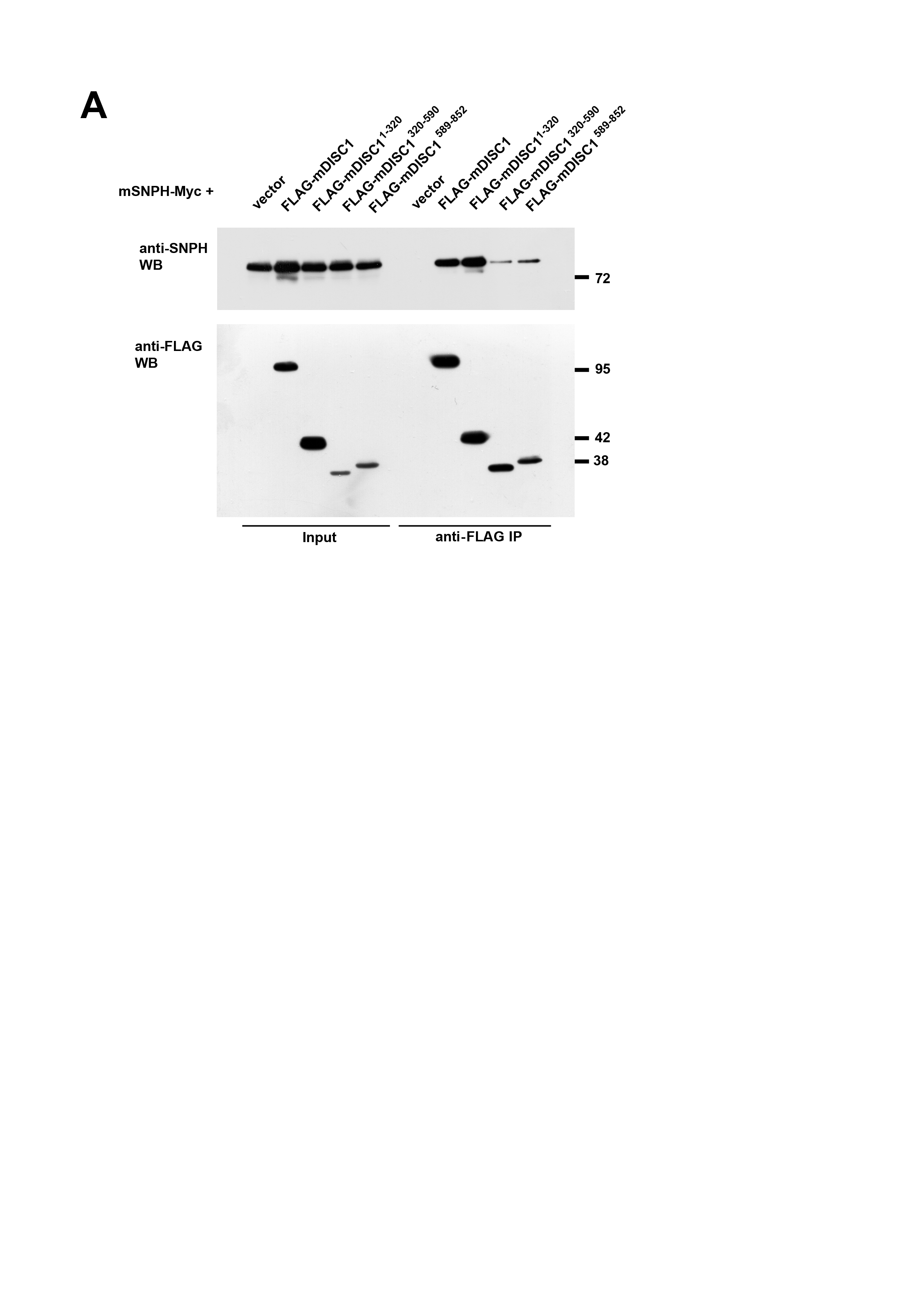

Supplement: Additional file 1: Figure S1. — Multiple SNPH association regions of DISC1. (A) Co-immunoprecipitation of FLAG-mDISC1 fragments with mSNPH-Myc in HEK293 cells. Lysates were immunoprecipitated with anti-FLAG and subjected to anti-FLAG and anti-SNPH western blotting. (TIF 1174 kb) [file 13041_2016_250_MOESM1_ESM.tif]

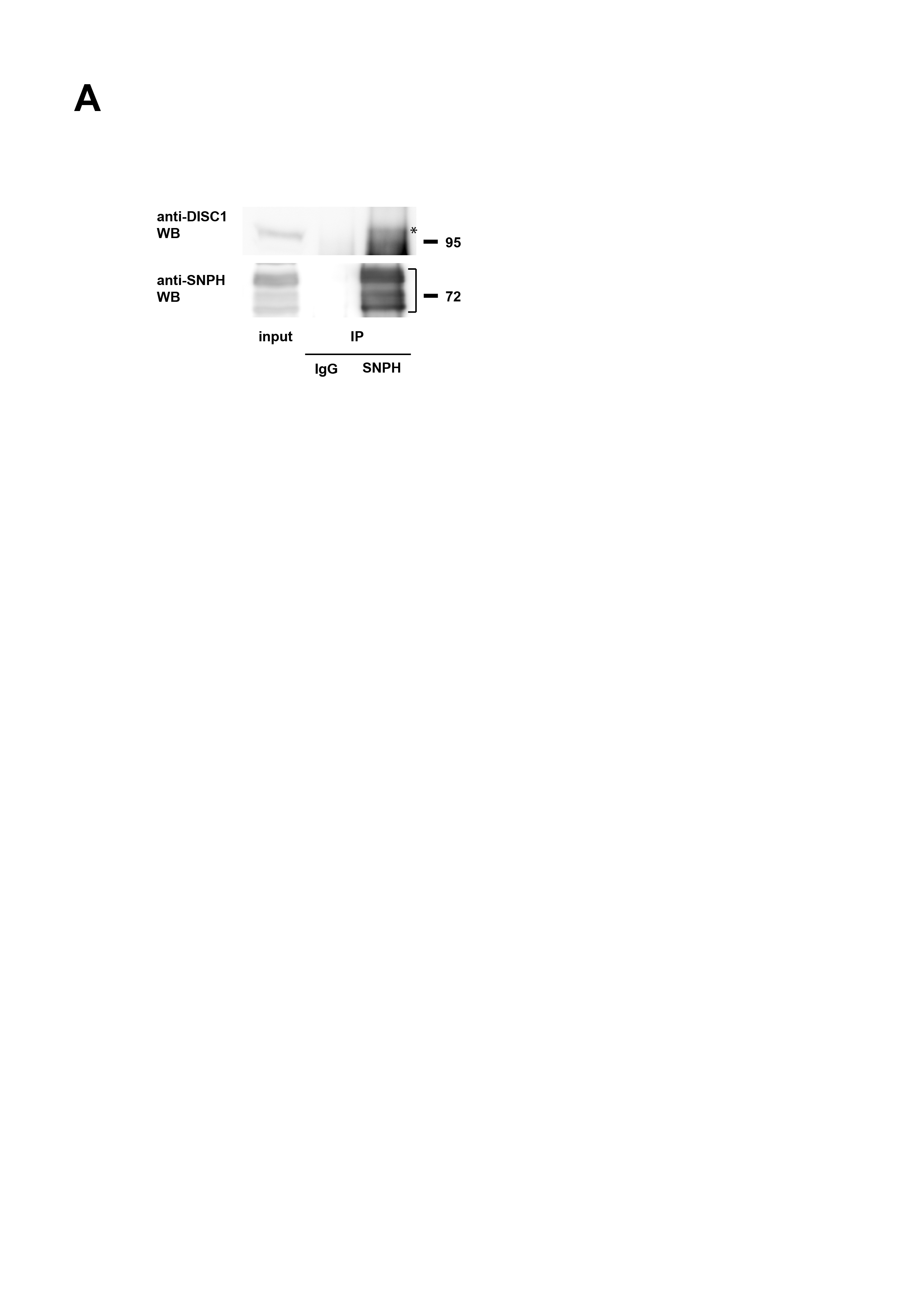

Supplement: Additional file 2: Figure S2. — DISC1 and SNPH association in the mouse whole brain tissue. (A) Co-immunoprecipitation of endogenous SNPH and DISC1 from the mouse brain lysates. Anti-SNPH immunoprecipitates were analyzed by western blotting with anti-SNPH and anti-DISC1 antibodies. The asterisk and bracket indicate endogenous DISC1 and SNPH, respectively. (TIF 674 kb) [file 13041_2016_250_MOESM2_ESM.tif]

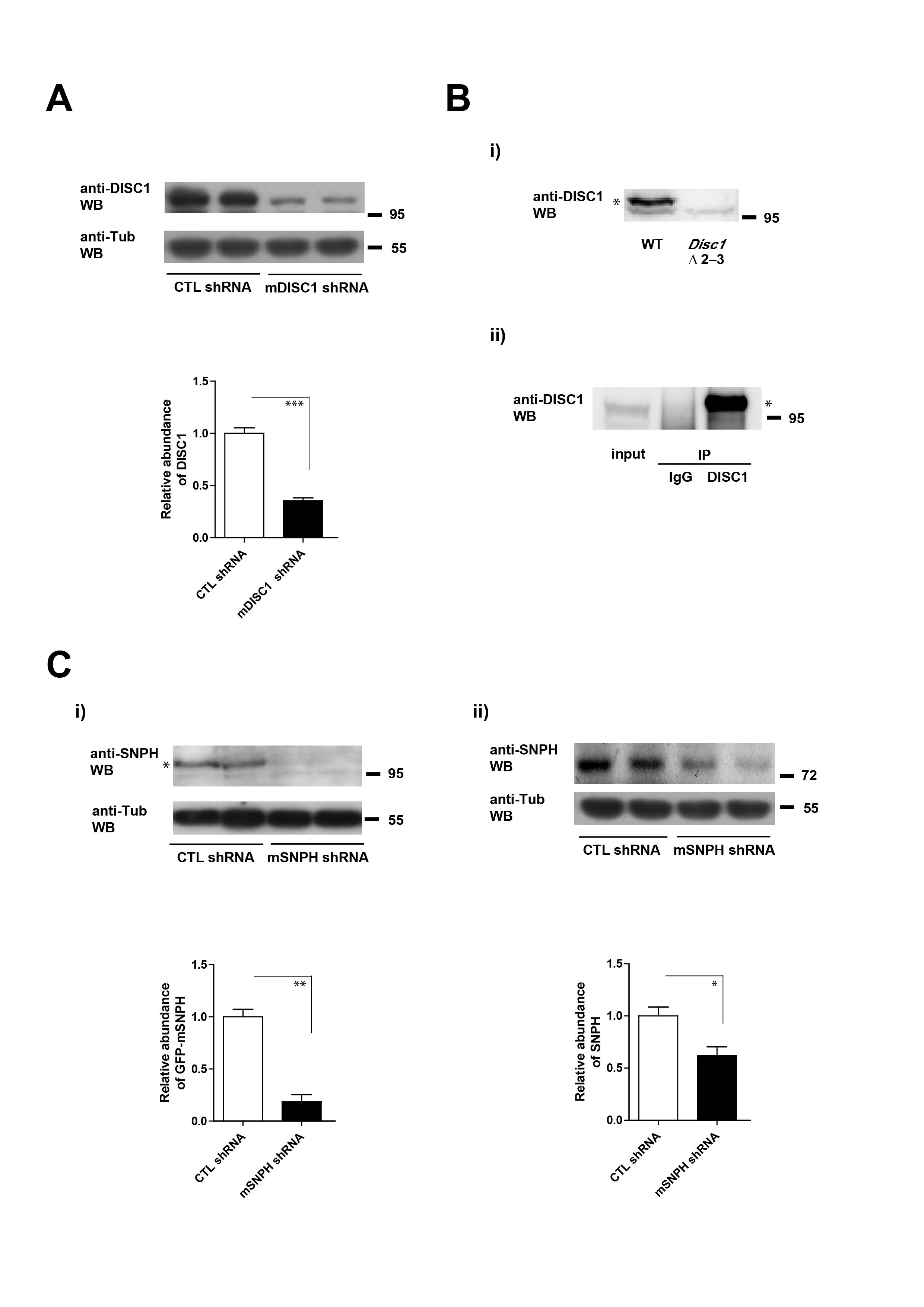

Supplement: Additional file 3: Figure S3. — Characterization of shRNA constructs and antibodies against DISC1 and SNPH. (A) Knockdown of endogenous DISC1 in the differentiated CAD cells using DISC1 shRNA. Quantification of band intensities is also shown. Error bars represent means ± SEM. *** P < 0.001 (student’s t-test, n = 3). (B) Characterization of DISC1 antibody in the whole brain lysates. (i) Detection of DISC1 in the brain lysates from the wild-type (WT) and mutant mice lacking exons 2 and 3 of Disc1 gene [Disc1 (∆ 2–3)]. (ii) The immunoprecipitated DISC1 from mouse brain lysates. The asterisk indicates the endogenous DISC1 band. (C) Knockdown of overexpressed GFP-mSNPH (i) and endogenous SNPH (ii) in the differentiated CAD cells using SNPH shRNA. GFP-mSNPH is indicated by a single asterisk in the western blotting. Quantification of band intensities is also shown. Error bars represent means ± SEM. *P < 0.05, **P < 0.01 (student’s t-test, n = 3). (TIF 1040 kb) [file 13041_2016_250_MOESM3_ESM.tif]

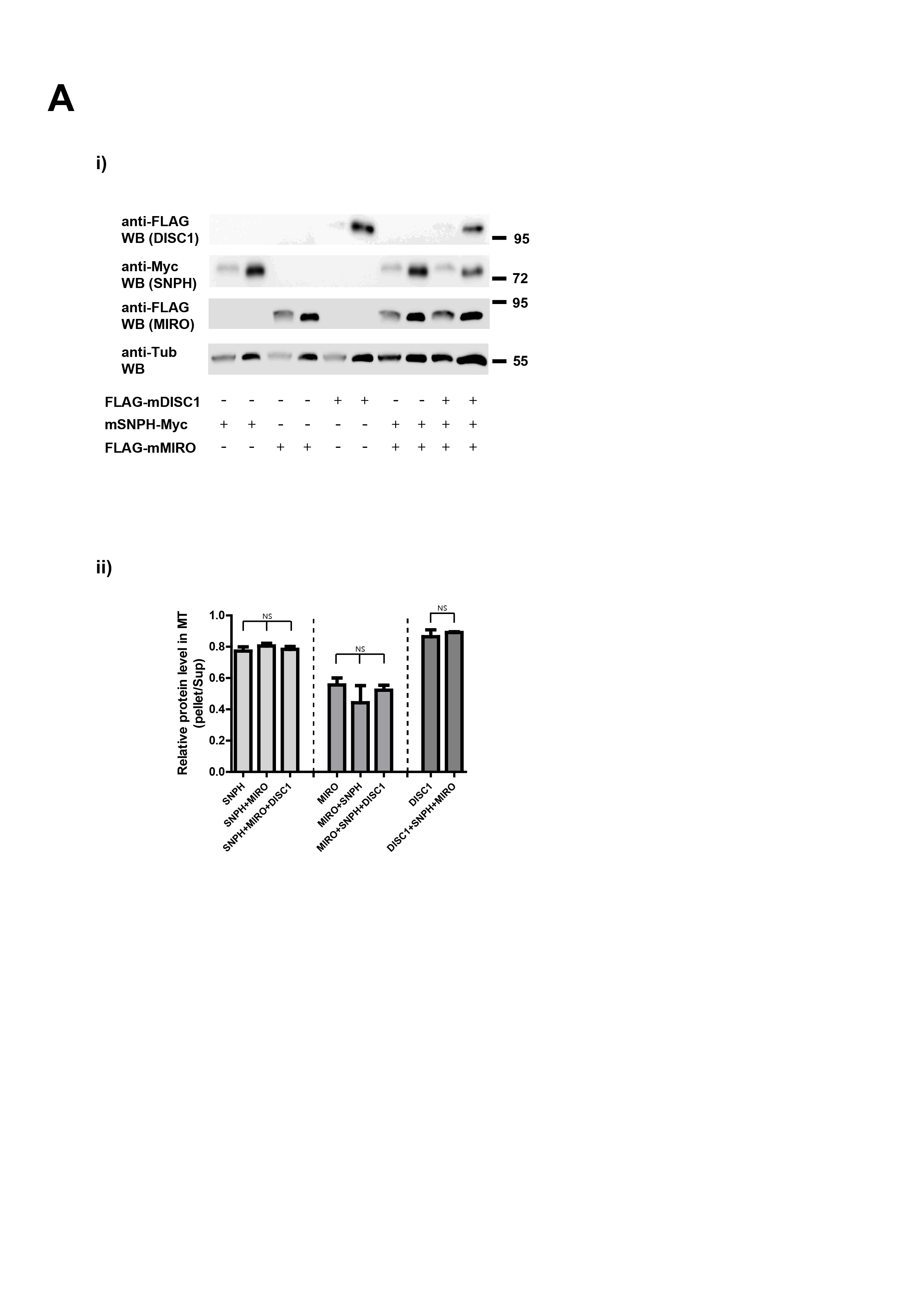

Supplement: Additional file 4: Figure S4. — Unaffected microtubule association of SNPH and MIRO upon DISC1 co-expression. (A) Microtubule co-sedimentation assay. HEK293 cell lysates transfected as indicated were incubated with 20 μM of Taxol for 30 min at 37 °C. Following the sedimentation of polymerized microtubule by centrifugation at 12,000 g for 40 min at room temperature, same amount of each sample were subjected to the (i) western blotting and (ii) densitometric analysis S; supernatant. P; pellet. (TIF 725 kb) [file 13041_2016_250_MOESM4_ESM.tif]
